# Etiopathogenesis and Therapy of Epithelial Ingrowth after Descemet's Stripping Automated Endothelial Keratoplasty

**DOI:** 10.1155/2014/906087

**Published:** 2014-09-02

**Authors:** Francesco Semeraro, Attilio Di Salvatore, Alessandro Bova, Eliana Forbice

**Affiliations:** Eye Clinic, Department of Neurological and Vision Sciences, University of Brescia, Piazzale Spedale Civili 1, 25123 Brescia, Italy

## Abstract

Descemet's stripping endothelial keratoplasty is an emerging technique finalized to treat endothelial dysfunction replacing only the pathological portion of cornea. The advent of any new technique puts us in front of new complications. The epithelial ingrowth is a well-known complication already studied in case of ocular trauma and more recently in refractive surgery. This job analyzed the potential etiopathogenesis of epithelial ingrowth after DSAEK, reviewing the cases described in literature, and suggests the potential therapy.

## 1. Introduction

In the past decade, Descemet's stripping automated endothelial keratoplasty (DSAEK) has become the chosen procedure for the management of patients with endothelial dysfunction, overtaking penetrating keratoplasty (PK) in popularity for the treatment of these specific diseases [1]. The technique was originally described by Melles et al., [2] as “posterior lamellar keratoplasty,” and was subsequently modified by Terry and Ousley [3] and renamed “deep lamellar endothelial keratoplasty.” The technique of stripping Descemet's membrane was again described by Melles et al. [4] and termed “Descemet's stripping endothelial keratoplasty.” The procedure was modified again to involve the use of an automated blade microkeratome to create a lamellar dissection of the donor cornea, as described by Gorovoy and Ratanasit [5], and was termed “Descemet's stripping automated endothelial keratoplasty” (DSAEK).

This technique has advantages such as minimal refractive changes and more rapid recovery compared to PK [6]. However, it is not free of complications, which include donor graft detachment, postoperative graft dislocation, pupillary block glaucoma, cataract development, aqueous misdirection syndrome, and epithelial ingrowth [7–10]. One of the most frequent DSAEK complications is the opacity that arises from interface abnormalities. Interface opacities are a well-documented phenomenon observed after DSAEK [10] caused by epithelial ingrowth, infection, inflammation, retention of Descemet's membrane, interface blood, calcium, interface fluid, retained viscoelastic material, or an irregular donor cut [11, 12]. Epithelial ingrowth has been reported to cause interface opacity within a lamellar graft after DSAEK [5, 8, 9, 11, 13–19].

Historically, epithelial ingrowth was described as an anterior chamber growth that rarely occurs after cataract extraction [20], penetrating keratoplasty (PKP) or other invasive ocular surgeries such as anterior chamber aspiration and glaucoma procedures [21]. In the recent literature, the most common experience with epithelial ingrowth into a lamellar interface follows laser in situ keratomileusis (LASIK). The reported incidence of intracorneal epithelial ingrowth after a corneal lamellar surgery such as LASIK is 20% [22, 23]. The reported frequency of epithelial ingrowth after DSAEK is very low.

Herein, we review the literature on epithelial ingrowth after DSAEK in order to better understand the risk factors, generation mechanisms, and potential therapy associated with this complication of surgery. The results of our literature search on epithelial ingrowth, epithelial interface implantation, and epithelial ingrowth after DSAEK are shown in Table 1.

Although many authors use the term “downgrowth” [5, 9, 13, 14, 19] to describe this phenomenon, we prefer, as other have suggested, the term “ingrowth” to describe this grow into the interface [8, 11, 17, 18, 24].

## 2. Etiopathogenesis, Risk Factors, and Mechanism

Epithelial ingrowth is characterized by the migration and growth of corneal or conjunctival epithelial cells into the anterior chamber of the eye through a breach in the ocular surface or into the lamellar interface of the cornea itself after corneal lamellar or flap surgery [8].

The ingrowth observed after Descemet's keratoplasty generally develops at the interface, between the patient's stroma and the donor's lamella. In these cases, early epithelial ingrowth is seen as a haze during the slit-lamp examination, with a sharp demarcation representing multiple layers of corneal epithelium (resembling normal corneal epithelium), suggesting considerable proliferation. The late stage appears clinically as a homogenous white mass, which comprises clumps containing amorphous materials with scarce cellular elements or cellular debris, suggesting less proliferation [25]. In more severe cases, epithelial ingrowth may extend from the interface to a retrocorneal membrane with extension onto the iris surfaces, causing ectropion uveae, corneal decompensation, and glaucoma [18, 21]. However, not every case of suspected epithelial implantation leads to graft failure, and interface epithelial inclusions can remain static or even regress over time [8, 13, 16, 18, 19, 26].

Several groups have investigated the etiology of epithelial cells. Three main mechanisms were proposed as causes of epithelial ingrowth after DSAEK:

1. dragging of loose epithelial cells intracamerally or onto the stromal surface during graft insertion [17, 19],
2. migration of epithelial cells from the donor epithelium on eccentrically trephined grafts containing full-thickness cornea [27],
3. introduction of epithelial cells from full-thickness cornel incisions (i.e., Venton incisions) [8].

Many reported cases of epithelial ingrowth involve intraoperative and/or postoperative complications (see Table 2). The first specific risk factor for the occurrence of epithelial ingrowth after DSAEK is graft dislocation or graft detachment, necessitating rebubbling or reattachment [8, 11, 14, 17, 19, 24]. Graft detachment/dislocation further exposes the area of denuded endothelium, which may facilitate the migration of loose epithelial cells. Seeded epithelial cells may proliferate within the denuded graft-host interface without the contact inhibition provided by endothelium [28, 29]. Reattachment procedures may subsequently trap the retained epithelial cells, allowing further proliferation at the interface. Another known risk factor is the combination of cataract extraction and IOL implantation along with DSAEK, which increases the amount of surgical manipulation and provides a portal of entry to the anterior chamber for host epithelial cells [5].

Wound leak and tissue incarceration are also considered risk factors for epithelial ingrowth; the presence of vitreous within the surgical wound can act as a scaffold for the migration of recipient epithelium [14, 21]. In a case report by Phillips et al. [14], histological examination of the failed DSAEK graft showed multilayered conjunctival epithelium; the ingrowth thus originated from the recipient conjunctiva. Possible causes of epithelial ingrowth in this case include the presence of vitreous within the surgical wound, which could provide a scaffold for the migration of conjunctival epithelium from the conjunctival tissue adjacent to a compromised wound. Furthermore, the location of the surgical incision may facilitate epithelial cell entry. Corneal or limbal incisions, as opposed to scleral tunnel incisions, allow loose epithelial cells at the cornea or limbus to be dragged and introduced into the anterior chamber, leading to epithelial ingrowth [18].

Preparation of the posterior lamellar disc manually or with use of a microkeratome is an important factor contributing to epithelial ingrowth. It is postulated that the donor epithelium may be implanted on the graft during preparation of the donor posterior lamellar disc and then introduced intraoperatively at the interface or in the anterior chamber [11, 16]. In a large series of patients with epithelial ingrowth Suh et al. [27] reported that epithelial cells originated from the full-thickness portion of the DSAEK graft after eccentric trephination of the donor tissue. In other cases, the donor epithelium was scraped off prior to use of the microkeratome during preparation of the DSAEK lenticule. Loose donor epithelial cells may thus be dragged across the stromal interface by the microkeratome and remain adherent to the stroma.

## 3. Diagnostic Procedures

**Figure 1:**
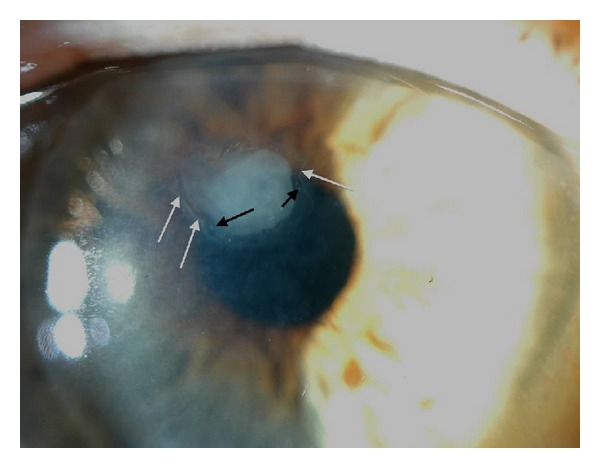
A case of epithelial ingrowth appeared two months after surgery. Epithelial pearls (black arrows) and a demarcation line (white arrow) could be seen.

During slit lamp biomicroscopy, epithelial ingrowth can appear as a flat haze that gradually increases in size with the development of epithelial pearls (see Figure 1), ultimately developing into a homogeneous whitish mass with a sharp demarcation, likely due to the fusion of epithelial pearls. Indirect slit-lamp illumination can sometimes help visualize this sheet-like proliferation. The use of fluorescein solution can also aid in diagnosis as well as in the prevention of postoperative recurrence by highlighting corneal abnormalities and epithelial fistulas, retracted tissue or an elevated wound edge.

**Figure 2:**
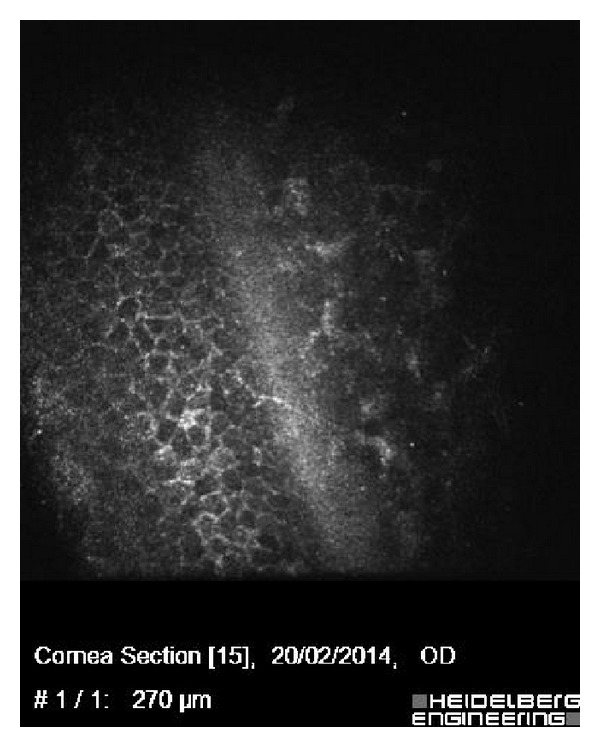
In vivo confocal micrographs showing microstructural changes (typically epithelial cells with prominent borders and distinctive nuclei) in the interface between the flap and stromal bed.

Confocal microscopy permits noninvasive, in vivo microscopic examination of all layers of the cornea [19]. In cases of epithelial ingrowth, this technique may reveal an epithelial cell-like mass (large, polygonal cells suggestive of epithelial elements) at the graft-host interface, forming clusters or nests within fibrotic tissue [30]. Thus, epithelial ingrowth can be identified and distinguished from fibrous proliferation (see Figure 2). Confocal microscopy may also prove useful in following the clinical course of epithelial ingrowth after treatment and may prove to be more sensitive than routine light microscopy for the detection of residual epithelial ingrowth [31, 32]. Prior to the advent of vivo confocal microscopy, the cellular changes associated with intraepithelial ingrowth were rarely described because PKP or flap removal was required for histological examination. We think that confocal microscopy may be the method of choice for evaluating epithelial ingrowth.

Through histopathology, scientists identified the extension of epithelium over donor endothelium as the cause of graft failure. The analysis of failed grafts after excision revealed epithelium on the posterior surface of the tissue [14]. XY karyotyping was performed to determine whether the tissue was of donor or host origins [5, 11, 19].

**Figure 3:**
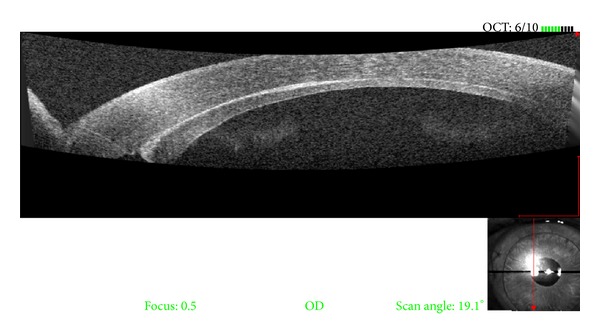
Optical coherence tomography (OCT) showing hyporeflective clefts and irregular, hyperreflective masses which may represent different layers of epithelium trapped at the interface between the flap and stromal bed in a case of epithelial ingrowth.

Optical coherence tomography (OCT) visualizes epithelial ingrowth at the interface as hyporeflective clefts and irregular, hyperreflective masses [18], which may represent different layers of epithelium trapped at the interface (see Figure 3). The predominance of hyporeflective clefts may represent the presence of basal epithelial layers, as would result from an early or arrested stage of epithelial ingrowth at the interface. These areas, however, may also represent fluid or debris trapped at the interface; histological confirmation will be necessary for conclusive evidence that these layers represent various layers of epithelium. In a series of 5 eyes from Suh et al. [18], OCT was used to study the origins of epithelial ingrowth. In Cases 2 and 4, the hyperreflective layer was found to be contiguous with the temporal incision. In case 3, the hyperreflective layer was continuous with a full-thickness edge of the DSAEK graft, representing an eccentric trephination of the donor tissue. Hyperreflective layers contiguous with the temporal incisions, as shown by OCT in Cases 2 and 4, may delineate the track through which epithelial cells gained access during graft insertion. In case 3, the epithelium was likely derived from the full-thickness edge of the DSAEK graft, a mechanism of epithelial ingrowth reported in prior histopathological studies [28] of failed DSAEK grafts. In case 5, the fistulous tract from the limbal incision was likely the entryway through which epithelial cells gained access to the eye [18].

Pentacam technology has been used to measure reflectivity at the interface region between the graft and the host cornea in optically clear corneas at various time points after DSAEK [33]. However, no such report on epithelial ingrowth has been published to date. Furthermore, despite the utility of topographical technology in assessing the effects of ingrowth on astigmatism, this technique is not used to evaluate DSAEK patients.

## 4. Pathophysiology and Treatment

This review highlights the role of epithelial cells in epithelial ingrowth in order to clarify the associated pathophysiology and optimize treatment. Most of the risk factors for this condition can be minimized by performing DSAEK compared with standard PKP. It is therefore tempting to dismiss incidents of epithelial ingrowth as “chance” occurrences related to a history of multiple intraocular surgeries, the presence of vitreous in the wound, or the incidental implantation of epithelial cells at the time of surgery—rather than to the DSAEK procedure specifically.

As previously pointed out, the most common experience of epithelial ingrowth extending to the lamellar interface occurs in LASIK patients. Most cases of epithelial ingrowth after LASIK are self-limiting [22, 25]. Likewise, some cases of DSAEK-related ingrowth appear not to progress and sometimes they may even lead to gradual resolution spontaneously [8, 12, 26] (Table 1). In a series of 118 DSAEK patients, Suh et al. [26] found one case of presumed epithelial implantation at the interface, which had been documented clinically and by anterior segment OCT. In another case series, the same authors described five additional cases of epithelial ingrowth after DSAEK [18]. None of these cases developed into graft failure or deteriorated visual acuity, so no treatment was administered.

Bansal et al. [8] reported a case of epithelial ingrowth after DSAEK with stromal puncture for phakic bullous keratopathy, which they treated conservatively. The central visual axis was clear, and the epithelial ingrowth had not progressed at the end of followup (13 months). Bansal et al. [8] justified this low tendency to progress with the probability that the cells already present at the interface have limited proliferating potential and die after a few mitotic divisions, leaving amorphous debris seen clinically as a homogeneous white mass. While an intact corneal endothelium normally inhibits epithelial migration through contact inhibition, the loss of this protective effect would allow for the extension of ingrowth to the endothelium. It should be pointed that in these cases, which did not involve flap removal, no histological diagnosis was possible.

However, corneal graft failures and cases of severe interface abnormality attributed to epithelial ingrowth have recently been reported. In these cases, treatment typically involves surgical graft resection [5, 9–11, 14, 16–19, 24, 34]. Ghosh et al. [16] report a case of histologically proven epithelial ingrowth at the interface of graft and host tissue that resulted in graft failure after uneventful DSAEK. The patient was treated successfully by stripping and careful aspirating the interface material, followed by a second DSAEK. Phillips et al. [14] reported a case of two failed DSAEKs where histological analysis of the failed graft after removal showed conjunctival epithelial cells over the surgical margin and even on the posterior surface. In both cases, a second DSAEK was performed. Lee et al. [24], in a retrospective histopathologic study of eight corneas, found one case of epithelial ingrowth at the interface resulting in graft failure. This case involved donor graft dislocation during the first DSAEK procedure. A second DSAEK was required after graft failure. Gorovoy and Ratanasit [5] documented one case of epithelial ingrowth that was confirmed histopathologically in a patient who had undergone DSAEK. In their case, the donor cells were growing along the iris as well as at the interface; the patient was treated with a repeat DSAEK, and no other treatment was necessary. Koenig and Covert [17] describe a case of epithelial ingrowth confirmed histopathologically after a rebubbling procedure for recurrent donor lenticule dislocation during DSAEK. Donor lenticule exchange, mechanical scraping, and irrigation-aspiration of the residual epithelial cells were performed, and a new graft was provided. Signs of epithelial ingrowth were not observed during the two-year followup. In each of the cases presented above, a repeat DSAEK was considered as sufficient treatment. No additional therapy or penetrating keratoplasty was necessary for resolution of the patient's ocular pathology. It should be noted that each of these cases involved a histological diagnosis of epithelial ingrowth.

However, in other published reports, a more invasive treatment approach was deemed necessary, and PKP was performed. Walker et al. [19] describe a case of epithelial ingrowth after DSAEK that required repositioning with an air bubble one week after surgery. Three months after DSAEK, multiple opacities were noted at the graft-host interface, and in vivo confocal microscopy revealed large, polygonal cells thought to be epithelial cells at the DSAEK interface. The patient underwent uneventful penetrating keratoplasty, and the diagnosis was confirmed histopathologically. Signs of recurrent epithelial ingrowth were not noted at the end of the 18-month followup.

Saelens et al. [11] documented epithelium-lined cysts at the interface after a DSAEK performed using tissue of donor origin, as revealed by XY karyotyping. The patient subsequently underwent PKP due to graft failure. Culbertson [13] documented a case of epithelial ingrowth treated with PKP using confocal microscopy. Prasher et al. [9] reported two cases of epithelial ingrowth after DSAEK. In the first, the ingrowth was limited to the endothelial surface of the donor cornea and was treated with a repeat DSAEK. In the second, interface epithelial ingrowth was histologically confirmed as the cause of graft failure in a patient treated with PKP.

The application of intracameral 5-fluorouracil (5-FU) is a conservative approach used rarely to treat epithelial ingrowth. In three cases of DSAEK reported by Lai and Haller, intracameral 5-FU was used safely to treat epithelial ingrowth and recalcitrant interface haze. Antimetabolites such as 5-FU inhibit cell proliferation, which allows the treatment to target epithelial cells in cases of ingrowth [35]. Wong et al. [36] describe the case of a 79-year-old woman who underwent DSAEK and subsequently presented with persistent interface haze. In this case, there was early evidence of a translucent membrane at the interface that extended over the peripheral iris inferotemporally. Argon laser photocoagulation was applied to the membranous growth, resulting in a whitening response characteristic of epithelial tissue. Epithelial ingrowth was diagnosed, and intracameral 5-FU was administered. One year after this single injection, the patient had a clear DSAEK graft without interface haze.

Aggressive surgical options may be considered in cases of epithelial ingrowth after DSAEK with extrainterface extension. Suh et al. [18] reported a case of epithelial ingrowth after DSAEK present at the interface and also as a retrocorneal membrane with extension onto the iris surface, causing ectropion uveae. This case of epithelial ingrowth was treated with block excision and corneoscleral grafting. However, Gorovoy and Ratanasit [5] suggested that the epithelial cells in patients who have undergone DSAEK cause less damage than expected. Observation may be indicated until symptomatic graft edema is accompanied by extensive diffusion. The authors go on to suggest a repeat DSAEK rather than PKP in cases of graft failure.

## 5. Conclusions

This review of the literature emphasizes that prevention is the mainstay treatment for epithelial ingrowth after DSAEK [37]. Prophylactic anterior vitrectomy should be performed in any case where posterior capsule integrity is in question and vitreous may be present in the anterior chamber. Wound construction and approximation with sutures should be prioritized, and excessive postoperative inflammation should be avoided. Every attempt should be made to avoid excessive endothelial cell damage, which might provide an easy path for the overgrowth of epithelial cells [14]. Extreme attention and meticulous technique are recommended at all stages of the DSAEK procedure [16]. Once intracorneal epithelial ingrowth is detected, careful evaluation by confocal microscopy and close followup are necessary.

In the case of progressive pathology leading to graft failure, early recognition, careful removal of the implanted epithelium, and repeat DSAEK may help achieve a successful outcome without the need for more invasive treatments [16]. Otherwise, disease progression can lead to severe opacity and graft failure involving the stroma, ultimately requiring PKP [9, 11, 13, 19]. However, it seems that even in the case of diffusion and extension over the interface observation is recommended over more aggressive approaches, because DSAEK-related ingrowth is less aggressive than commonly assumed. Even in severe cases, observation may be indicated until the appearance of symptomatic graft edema [5, 14].

Antimetabolites such as 5-FU may be used alone as a conservative approach to therapy.

In any case, a careful and noninvasive approach is advised. This stands in contrast with the common approaches taken by surgeons in case of ingrowth in penetrating surgery (PK, glaucoma, and cataracts) that frequently results in extending pathology requiring early and aggressive treatment.

## Figures and Tables

**Table 1:** Literature review of epithelial migration into anterior chamber after DSAEK.

Study	Numeber of eye(s)	Description	Diagnosis	Graft failure	Treatment
Culbertson [13]	1	Epithelial downgrowth	Confocal microscopy	No	PK

Koenig and Covert [17]	1	Epithelial ingrowth,interface	Histology	Yes	Repeat DSAEK

Walker et al. [19]	1	Epithelial downgrowth, at the interface	Confocal microscopy, histology	No	PK

Prasher et al. [9]	2	Case 1-epithelial downgrowth, interface	Histology	Yes	Case 1 had PK
		Case 2-epithelial downgrowth, not at the interface			Case 2 had DSAEK

Phillips et al. [14]	1	Conjunctival epithelial downgrowth, over donor endothelium	Histology	Yes	Repeat DSAEK

Gorovoy and Ratanasit [5]	1	Epithelial downgrowth, not at the interface	Histology	Yes	Repeat DSAEK

Saelens et al. [11]	1	Epithelial ingrowth in the flap-graft interface	Histology		Posterior mushroom keratoplasty

Lee et al. [24]	1	Epithelial ingrowth at the interface	Histology	Yes	Repeat DSAEK

Suh et al. [18]	5	Epithelial ingrowth interface-1	AS-OCT-1, spectral domain	None documented	Observation in 4 cases
		Interface retrocorneal-4	Ultrahigh resolution OCT-3, histology-1		Corneoscleral grafting in one case

Bansal et al. [8]	1	Epithelial ingrowth after stromal puncture	Clinical	No	Nil

Ghosh et al. [16]	1	Epithelial ingrowth interface	Histology	Yes	Repeat DSAEK

Wong et al. [34]	1	Interface haze (atypical presentation of epithelial ingrowth interface)	Slit lamp, translucent membrane whitened after argon laser photocoaugulation	Yes	3 DSAEKs intracameral 5-FU

DSAEK: Descemet stripping automated endothelial keratoplasty; PK: penetrating keratoplasty; AS-OCT: anterior segment optical coherence tomography; OCT: optical coherence tomography.

**Table 2:** Risk Factors of epithelial ingrowth.

Risk factors	Mechanism of ingrowth	Authors
Graft dislocation or graft detachment	Exposition of denuded endothelium areas, probable loss of the contact inhibition provided by the endothelium. Proliferation and migration of loose epithelial cells	Bansal et al. [8], Saelens et al. [11], Koenig and Covert [17], Phillips et al. [14], Walker et al. [19], Lee et al. [24], Sidrys and Demong [28], Cameron et al. [29]

Combination of cataract extraction and IOL implantation	Surgical manipulations may provide a portal of entry for host epithelial cells into the AC.	Gorovoy and Ratanasit [5]

Wound leak or tissue incarceration	Presence of vitreous within the surgical wound as a scaffold for the epithelial conjunctival cells migration, loss of endothelium cells inhibition	Phillips et al. [14], Chen and Pineda II [21]

Location of the surgery incision	Limbal or corneal incision would facilitate near loose epithelial cells to be dragged and introduced into the anterior chamber	Suh et al. [18]

Preparation of the posterior lamellar disc	The donor epithelium can be implanted on graft during the preparation of the donor posterior lamellar disc and then introduced intraoperatively at interface of AC. The loose donor epithelial cells may be mechanically dragged across the stromal interface by microkeratome and remain adherent to the stroma, developing epithelial ingrowth	Saelens et al. [11], Ghosh et al. [16], Suh et al. [27]
